# Clusters of the Risk Markers and the Pattern of Premature Coronary Heart Disease: An Application of the Latent Class Analysis

**DOI:** 10.3389/fcvm.2021.707070

**Published:** 2021-12-08

**Authors:** Leila Jahangiry, Mahdieh Abbasalizad Farhangi, Mahdi Najafi, Parvin Sarbakhsh

**Affiliations:** ^1^Tabriz Health Services Management Research Center, Tabriz University of Medical Sciences, Tabriz, Iran; ^2^Health Education and Health Promotion Department, School of Health, Medical Education Research Center, Health Management and Safety Promotion Research Institute, Tabriz University of Medical Sciences, Tabriz, Iran; ^3^Department of Community Nutrition, Faculty of Nutrition, Tabriz University of Medical Sciences, Tabriz, Iran; ^4^Department of Anesthesiology, Tehran Heart Center, Tehran University of Medical Sciences, Tehran, Iran; ^5^Schulich School of Medicine and Dentistry, Western University, London, ON, Canada; ^6^Department of Epidemiology and Biostatistics, Faculty of Health, Tabriz University of Medical Sciences, Tabriz, Iran

**Keywords:** coronary heart diseases, risk markers, premature, cluster analysis, gender

## Abstract

**Background:** Coronary heart disease (CHD) is the major cause of mortality in the world with a significant impact on the younger population. The aim of this study was to identify prematurity among patients with coronary artery bypass graft surgery (CABG) based on the clustering of CHD risk factors.

**Methods:** Patients were recruited from an existing cohort of candidates for CABG surgery named Tehran Heart Center Coronary Outcome Measurement (THC-COM). A latent class analysis (LCA) model was formed using 11 potential risk factors as binary variables: cigarette smoking, obesity, diabetes, family history of CHD, alcohol use, opium addiction, hypertension, history of stroke, history of myocardial infarction (MI), peripheral vascular disease (PVD), and hyperlipidemia (HLP). We analyzed our data to figure out how the patients are going to be clustered based on their risk factors.

**Results:** For 566 patients who were studied, the mean age (SD) and BMI of patients were 59.1 (8.9) and 27.3 (4.1), respectively. The LCA model fit with two latent classes was statistically significant (*G2* = 824.87, *df* = 21, *p* < 0.0001). The mean (SD) age of patients for Class I and Class II was 55.66 (8.55) and 60.87 (8.66), respectively. Class I (premature) was characterized by a high probability of smoking, alcohol consumption, opium addiction, and a history of MI (*P* < 0.05), and class II by a high probability of obesity, diabetes, and hypertension.

**Conclusion:** Latent class analysis calculated two groups of severe CHD with distinct risk markers. The younger group, which is characterized by smoking, addiction, and the history of MI, can be regarded as representative of premature CHD.

## Highlights

- Clustering of patients with coronary artery diseases provide a better understanding of the disease behavior.
- Clustering patients based on individual and clinical characteristics help health counselors to predict premature CHD.
- Findings are applicable to policy makers and health professionals for coronary artery diseases (CHD) management.

## Background

Coronary heart disease (CHD) is the major cause of mortality in the world and one of the leading causes of disease burden in developing and developed countries (1). Of the 17 million deaths from cardiovascular diseases (CVDs) in 2015, 7.4 million people died of CHD. It is responsible for over a third of premature deaths globally (1). The number of CABG operations performed to treat CHD has increased more than five-fold since 1980, and the general trend has been an almost steady rise in the number of operations performed each year (2, 3). With increasing the number of patients undergoing CABG, their healthcare costs have quadrupled in the last decade (4, 5).

There is no consensus on the definition, risk factors, and age limits for premature CHD in the clinical setting. The Task Force of the European Society of Cardiology (ESC), European Atherosclerosis Society (EAS), and European Society of Hypertension (ESH) have recommended screening for coronary risk factors to the close relatives of patients with premature CHD. They have been defined premature CHD as having CHDs for men <55 years and women <65 years (6). Sutter et al. have employed this definition to identify premature CHD in their study (7). Some have proposed <51 years for men and <56 for women as a cutoff point for premature CHD (8). Mulders et al. used this definition to classify the patients and showed that patients with a genetic predisposition for CVD are at risk for recurrent events (9). However, the age limit as low as 55 years for women and 45 years for men was suggested as well (10). Clinicians need a definitive definition as well to identify patients with premature CHD and prevent any misdiagnosis in the clinical setting.

Latent class analysis (LCA) is a commonly used empirical approach for identifying subpopulations by shared item response patterns. It is also a commonly used data reduction tool for analyzing multivariate categorical data (11). A finite number of exclusive classes of individuals categorized as latent variables are assumed to compose this population (12). The LCA is a statistical procedure that allows the researcher to identify groups of individuals (or clusters) due to their differences within a dataset that would otherwise not be apparent. Traditional cluster and factor analyses are designed for continuous variables, but LCA is suitable for discrete and dichotomous variables. Awareness of these clusters may provide us with a better understanding of the disease behavior and identify patients at higher risk of morbidity (13, 14). Hence, LCA sounds like an appropriate approach for classifying patients according to their individual and clinical characteristics to predict premature CHD. This study was aimed to identify latent classes of patients with severe CHD who were candidates for CABG based on the clustering of proposed risk markers for premature CHD.

## Methods

### Participants

Participants in this cross-sectional study were candidates for CABG surgery recruited for Tehran Heart Center Coronary Outcome Measurement (THC-COM) study. Baseline data were collected as part of the large study started in May 2006 (15). The sample size calculation, recruitment method, and definitions have been explained elsewhere (16). Briefly, a total of 535 patients were recruited to this study. Based on numerous studies, Nylund-Gibson and Choi suggest that 300 or more cases are desirable for sampling (17). Written informed consent was obtained from each participant. This study was approved by the Institutional Research Board of Tehran Heart Center, Tehran University of Medical Sciences.

### Anthropometric, Clinical, and Laboratory Measurements

Data collection was conducted through interviews, physical examinations, and paramedical evaluation. Patients were asked about demographic characteristics, including gender, age, family history of CHD [a first-degree relative with CHD was defined as positive family history (18)], cigarette smoking (patient regularly smokes a tobacco product/products one or more times per day or has smoked in the 30 days prior to admission) (19), alcohol consumption (the use of alcohol despite recurrent adverse consequences) (20), drug addiction (opium dependence according to the DSM-IV criteria for substance dependence, daily regular use of substances) (12), and having diabetes mellitus [symptoms of diabetes plus at least one of the following: plasma glucose concentration = 200 mg/dl (11.1 mmol/l), fasting plasma glucose = 126 mg/dl (7.0 mmol/l), plasma glucose 2 h postprandial = 200 mg/dl (11.1 mmol/l)] (21), and hypertension (systolic blood pressure = 140 mmHg and/or diastolic = 90 mmHg and/or on antihypertensive treatment) (22). Anthropometric variables, including weight and height, were measured, and BMI was calculated (23). Weight was measured while patients wearing light clothes. Total cholesterol, HDL cholesterol (HDL-C mg/dl), triglycerides (TG mg/dl), and LDL cholesterol (LDL-C mg/dl) were measured. They were also evaluated for having a history of stroke, myocardial infarction (MI), peripheral vascular disease (PVD), and hyperlipidemia (HLP).

### Statistical Analysis

The continuous variables were compared using a *t*-test, and categorical variables were analyzed using a chi-square test between male patients and female patients. An LCA model was built using 11 known and proposed risk factors for CHD as binary variables: cigarette smoking, obesity, diabetes, family history of CHD, alcohol consumption, opium addiction, hypertension, PVD, HLP, history of stroke, and MI. The included risk factors were added to the statistical model based on premature systematic review studies (24, 25). The LCA analyses were conducted using the “poLCA” package in R 3.4.2. The LCA outcomes include the number of latent classes, the probability of each indicator in each class, and the classification of individuals based on their most likely latent class membership. The assumption underlying the LCA model is that the conditional response probabilities for each individual are to be the same. Also, the assumption of conditional independence declares that within each class, the indicators are independent of one another. The data were checked to ensure that the mentioned assumptions were established. It was assumed that within latent classes, manifest variables are independent of each other. Unmodeled dependence among indicators may induce poor model fit and incorrect values of information criteria [e.g., Bayesian information criterion (BIC)], Akaike information criterion (AIC), resulting in spurious latent classes (usually with an overestimated number of classes). Because our results had reasonable values of information criteria, we can conclude that in our data, the assumption of conditional independence and conditional-response probability in LCA was established.

There are various iterations for the number of identified latent classes. Latent class analysis determines the best model by comparing the frequencies of the observed and expected response patterns, and it calculates *G2*. For comparing the relative fit of the model according to the different number of classes, *G2* (likelihood ratio chi-square) is used. Then, AIC and BIC can be calculated for model selection. The smaller the value of AIC and BIC, the more fit the model. The fit model contains the optimum number of latent classes. Then, we can nominate these classes based on their characteristics (26, 27). In this analysis, expectation-maximization and Newton–Raphson algorithms were used to find the maximum-likelihood estimates of the model parameters (28).

## Results

### Patients' Characteristics

The patients' clinical characteristics are presented in Table 1. The mean age (SD) and BMI of patients were 59.1 (8.9) and 27.3 (4.1), respectively. Women had a significantly greater BMI and higher fasting blood sugar (FBS), blood urea nitrogen (BUN), cholesterol, and LDL-C compared to men. Hematocrit and the level of creatinine were significantly higher in men. The prevalence of major CHD risk factors among the patients is presented in Table 2. Smoking was more prevalent among men, while diabetes and hypertension rates were significantly higher in women.

**Table 1:** Demographic, clinical, and preclinical parameters of the patients.

	**Total (*n* = 535)**	**Male (402)**	**Female (*N* = 133)**	***p*-Value**
Age (Mean; SD)	59.1 (8.9)	58.9 (9.4)	59.7 (7.4)	0.386
BMI (kg/m^2^)	27.3 (4.1)	26.6 (3.5)	29.5 (4.5)	<0.0001
ALB (mg/dl)	4.6 (0.3)	4.6 (0.34)	4.5 (0.29)	0.194
Last FBS (mg/dl)	107.3 (35.3)	103.9 (32.0)	117.7 (42.3)	<0.0001
HCT (mg/dl)	42.2 (4.1)	43.1 (4.0)	39.6 (3.1)	<0.0001
Creatinine (mg/dl)	1.3 (0.27)	1.3 (0.27)	1.1 (0.2)	<0.0001
BUN (mg/dl)	39.8 (12.1)	39.6 (12.0)	40.3 (12.4)	0.599
TG (mg/dl)	171.7 (88.6)	169.6 (93.7)	178.0 (71.2)	0.347
Cholesterol (mg/dl)	160.0 (45.1)	155.1 (41.7)	175.1 (51.3)	<0.0001
LDL-C (mg/dl)	86.4 (38.8)	84.0 (37.7)	93.6 (41.1)	0.013
HDL-C (mg/dl)	40.3 (8.4)	39.2 (8.1)	43.7 (8.7)	<0.0001
CRP (mg/dl)	7.0 (5.8)	6.9 (5.1)	7.3 (7.6)	0.547
Number of grafts	3.8 (3.3)	3.8 (0.9)	4.0 (6.5)	0.553
Smoking	188 (35.1)	183 (45.5)	5 (3.8)	<0.0001
Obesity	381 (71.2)	271 (67.4)	110 (82.7)	<0.0001
Diabetes	221 (41.3)	139 (34.6)	82 (61.7)	<0.0001
Family history	258 (48.2)	189 (47.0)	69 (51.9)	0.368
Alcohol consumption	72 (13.5)	72 (17.9)	0 (0.0)	<0.0001
Opium addiction	77 (14.4)	75 (18.7)	2 (1.7)	<0.0001
Hypertension	264 (49.3)	167 (41.5)	97 (72.9)	<0.0001
History of stroke	22 (4.1)	16 (4.0)	6 (4.5)	0.802
MI history	276 (51.6)	226 (56.2)	50 (37.6)	<0.0001
PVD history	157 (29.3)	96 (23.9)	61 (45.9)	<0.0001
HLP	378 (70.7)	259 (64.4)	119 (89.5)	<0.0001

*BMI, body mass index; ALB, albumin; FBS, fast blood sugar; HCT, hematocrit; BUN, blood urea nitrogen; TG, triglycerides; LDL-c, low-density lipoprotein; HDL-c, high-density lipoprotein; CRP, C-reactive protein; HLP, hyperlipidemia; PVD, peripheral vascular disease*.

**Table 2:** The latent class analysis and the calculated model for the prevalence of the indicators.

	**Premature** **class I**	**Non-premature** **class II**	***P*-value**
Latent class prevalence	0.338	0.662	
Items			
Smoking	**0.984**	0.068	<0.0001
Family history	**0.491**	0.478	0.855
Alcohol consumption	**0.386**	0.022	<0.0001
Opium addiction	**0.383**	0.037	<0.0001
History of MI	**0.679**	0.443	<0.0001
Obesity	0.626	**0.750**	0.003
Diabetes	0.318	**0.455**	0.007
Hypertension	0.347	**0.559**	<0.0001
History of stroke	0.039	**0.042**	0.838
PVD	0.272	**0.303**	0.671
HLP	0.675	**0.720**	0.705

*MI, myocardial infarction; PVD, peripheral vascular disease; HLP, hyperlipidemia. The values form the probabilities of the basis for interpreting and labeling the latent classes. They shows the conditional probabilities of having risk factor for each groups*.

### Latent Class Findings

Latent class membership and response probabilities for each indicator are summarized in Table 2. A model with two latent classes was the best fit for our patients (*G2* = 824.87, *df* = 21, *p* < 0.0001). The probability of membership was 33.8 and 66.2% for Class (I) and Class (II), respectively. Latent Class I was characterized by a high probability of smoking, a history of MI, alcohol consumption, and opium addiction (*P* < 0.05). Latent class II was characterized by a high probability of obesity, type 2 diabetes, and hypertension (*P* < 0.05). Family history, stroke, PVD, and HLP were not significantly different between the two classes. The models with 2, 3, 4, and 5 latent classes are shown in Supplementary Table 1.

Latent class membership and response probabilities for each indicator for men and women are presented in Table 3. The model indices showed that it is statistically significant for both men and women (*G2* = 731.7, *df* = 379, *p* < 0.0001 for men and *G2* = 162.5, *df* = 112, *p* < 0.0001 for women). Larger conditional probabilities are in boldface to highlight the overall pattern. While latent class I was characterized by a high probability of smoking, alcohol consumption, opium addiction, and a history of MI in men, it was characterized by a history of MI among women.

**Table 3:** The latent class analysis and the calculated model for the prevalence of the indicators among men and women.

	**Men**			**Women**		
	**Premature class I**	**Non-premature class II**	***P*-values**	**Premature class I**	**Non-premature class II**	***P*-values**
Latent class prevalence	0.455	0.545		0.518	0.482	
Smoking	**1.000**	0.000	<0.0001	**0.062**	0.014	0.547
Family history	**0.485**	0.458	0.775	0.000	**1.000**	<0.0001
Alcohol consumption	**0.363**	0.036	<0.0001	–	–	–
Opium addiction	**0.356**	0.054	<0.0001	**0.031**	0.000	0.302
History of MI	**0.672**	0.476	<0.004	**0.421**	0.333	0.004
Obesity	0.637	**0.702**	0.366	0.781	**0.869**	0.131
Diabetes	0.337	**0.352**	0.643	0.578	**0.652**	0.242
Hypertension	0.352	**0.464**	0.111	**0.781**	0.681	0.106
History of stroke	0.038	**0.040**	0.952	**0.093**	0.000	0.101
PVD	**0.275**	0.210	0.743	0.343	**0.565**	<0.0001
HLP	**0.689**	0.609	0.235	0.875	**0.913**	0.238

*MI, myocardial infarction; PVD, peripheral vascular disease; HLP, hyperlipidemia. The values form the probabilities of the basis for interpreting and labeling the latent classes. They show the conditional probabilities of having risk factor for each groups*.

The mean (SD) age for classes I and II was 55.66 (8.55) and 60.87 (8.66), respectively. There were significant differences in the age of the two groups of patients (*P* < 0.001). Patients in class I were about 5 years younger than patients in class II. Therefore, we named class I “premature” and class II “non-premature.” The rate of membership in class I for men and women was 44.3 and 2.7%, respectively. Also, the mean age (SD) of people who were categorized in class I was 55.58 (8.57) for men and 60 (6.55) for women. Only three women were categorized as class I or premature.

## Discussion

In this cross-sectional study, using LCA, two clusters of patients with severe CHD were identified with respect to 11 CHD risk markers. Class I is characterized by lower age, higher probability of smoking, alcohol consumption, opium addiction, and history of MI. Hence, we nominated class I as premature and class II as non-premature CHD. There was a difference between men and women with regard to the probability of their risk markers in the two classes.

There was a significant difference in the age of participants among the two classes. Class I included patients with younger age was named premature, and class II was named non-premature. A study used predefined criteria for addressing prematurity based on recommendations of other studies (7) before the age of 55 for men and 65 years for women (29). We employed LCA for clustering risk factors among CABG patients with similar results (55 years for men and 60 years for women) (Table 4). Some studies have shown that the risk of CHD increases with age >45 years in men and >55 years in women (7, 30). Another study applied the parental premature CHD definition when CHD occurred before 55/65 years in the father/mother patients with metabolic syndrome (8), suggesting that metabolic syndrome risk factors play a role in familial patterns for premature CHD.

**Table 4:** Mean (SD) of age according to sex and prematurity status.

	**Male *n* = 402 (75.14)**	**Female *n* = 133 (24.86)**
	**Age mean (SD)**	**Age mean (SD)**
Premature	55.58 (8.57)^*^	60.0 (6.55)
Non-premature	61.55 (9.2)^*^	59.68 (7.48)

* *P-value < 0.001*.

Many studies have addressed the differences between men and women with regard to the risk factors for premature CVD (16, 31). A study from Iran reported the incidence rate of premature CVD about five and four percent for men and women, respectively (32). A similar study from Iran revealed that modifiable lifestyle risk factors, hypercholesterolemia, low HDL-C, and diabetes were associated with premature CHD in both women and men patients (31, 33). In the recent study, being overweight, having less physical activity, and prediabetes were specific risk factors for premature CHD among women and smoking, and low HDL-C were associated with premature CHD among men. The current study used LCA and clustered 11 risk markers in two distinct class I (premature 33.8%) and class II (non-premature: 66.2%) groups in patients with severe CHD. Class I (premature) was characterized by the high prevalence of smoking (98.4%), alcohol consumption (38.6%), and opium addiction (38.3%). While in the other class, older patients were people with clinical risk factors, including diabetes (45.5%), obesity (75%), and hypertension (55.9%). Although positive family history has been reported as an important predictor for premature CHD (29, 30), we did not find such a relationship.

The known risk factors (such as diabetes, hypertension, dietary factors, obesity) and lipoprotein-associated risk (low HDL-C levels, higher TGs, and elevated apolipoprotein B levels) could explain premature CAD among South Asians (34). Studies have shown that opium addiction aggravates the known risk factors for CVD and has been considered an independent predictor for CHD mortality (8, 34, 35). According to a clinical study in Iran, about 5% of patients with CABG use opium. There is evidence that Iranian patients have a misconception of opium consumption that opium may decrease the risk of diabetes and hypertension (36). Similar studies determined that opium consumption decreases the age for MI and predisposes to cardiac death (37, 38).

To have a better knowledge of premature CHD risk factors, simultaneous classification of patients according to their conditions appears to be an important contributor.

Our findings have important implications for clinical practice and in particular, underline the importance of screening in lower age populations who have risk markers for premature CHD to adopt prevention strategies in primary and secondary care settings. This may be of benefit in the larger group of patients compared to those looking for a family history of CHD.

## Strengths and Limitations

We used LCA for distinguishing the pattern of risk factor distribution among our patients, and it worked well. A model of two groups fitted best and was in accordance with two age groups with the significantly different incidence of risk factors. However, there are some points to be addressed. The prevalence rates of smoking, alcohol consumption, and opium addiction were lower among women compared to those among men in our study. Latent class analysis categorized only three women in class I (premature CHD). A larger sample size is probably needed before we can judge the predictors and cutoff point for the age of premature CHD in women. Given the low rate of smoking, alcohol consumption, and opium addiction among women in studies (32), it is probable that CHD in younger women is represented by different determinants like PVD.

The average age of 55 years for men and 60 years for women that we found in our study is related to the Iranian patients with severe CHD who were candidates for CABG, and it cannot be applied for the diagnosis of premature CHD in other groups of patients. Moreover, we did not ask the patients about the time they were diagnosed as patients with CHD even though it is not practical to document the exact time the CHD has begun. However, coronary artery involvement was severe among all participants of this study and they were candidates for CABG, which means they were at the same level regarding the progress of CHD. Additionally, the cross-sectional design of this study restricts causal interpretation of the association between unhealthy behaviors in the development of CHD. The current study was the dataset of the THC-COM study, and it was not possible for authors to include other variables such as the duration of the study.

## Conclusion

In this study, we employed LCA to identify clusters of patients with regard to their risk markers and recognize different categories of CHD. The best model fitted two classes that we named premature and non-premature CHD. The latent class I (premature CHD) was characterized by smoking, alcohol consumption, opium addiction, and the history of MI, while class II was characterized by known major risk factors like obesity, diabetes, and hypertension. There was a difference between men and women regarding CHD risk markers.

## Data Availability Statement

The original contributions presented in the study are included in the article/Supplementary Material, further inquiries can be directed to the corresponding author/s.

## Ethics Statement

The studies involving human participants were reviewed and approved by Tehran University of Medical Sciences. The patients/participants provided their written informed consent to participate in this study. The study was approved by the Ethics Committee of Tehran University of Medical Sciences and Tabriz University of Medical Sciences (NO. IR.TBZMED.REC.1400.748). All participants gave written informed consent. The data and material is available.

## Author Contributions

LJ collected the data and wrote the first draft. MN was the main investigator who contributed to designing and helping in recruitment. MA helped in the interpretation of results and manuscript drafting. PS conducted study analysis. All the authors read and approved the manuscript.

## Conflict of Interest

The authors declare that the research was conducted in the absence of any commercial or financial relationships that could be construed as a potential conflict of interest.

## Publisher's Note

All claims expressed in this article are solely those of the authors and do not necessarily represent those of their affiliated organizations, or those of the publisher, the editors and the reviewers. Any product that may be evaluated in this article, or claim that may be made by its manufacturer, is not guaranteed or endorsed by the publisher.

## Abbreviations

BMI: body mass index
CVD: cardiovascular diseases
CABG, LCA: latent class analysis
MI: myocardial infarction
CHD: coronary heart disease
PVD: peripheral vascular disease
HLP: hyperlipidemia.
