# Supplementary material for: Clusters of the Risk Markers and the Pattern of Premature Coronary Heart Disease: An Application of the Latent Class Analysis
Source: Front Cardiovasc Med. 2021 Dec 8;8:707070. doi: 10.3389/fcvm.2021.707070 (PMC8692946; doi:10.3389/fcvm.2021.707070)
Supplement: Supplementary file 1 [file Table_1.docx]

Supplementary Table 1: The latent class analysis and the calculated model indicators for 2-5 classes

| K | AIC | BIC | G2 | Chi-square |
| --- | --- | --- | --- | --- |
| 2 | 6426.94 | 6525.44 | 824.87 | 2122.86 |
| 3 | 6377.60 | 6527.48 | 751.52 | 2126.463 |
| 4 | 6369.83 | 6571.10 | 719.76 | 2120.89 |
| 5 | 6365.89 | 6618.54 | 691.81 | 2138.95 |
